# Supplementary material for: Green synthesis and characterization of zinc chitosan nanoparticles with their anti-bacterial study against rice pathogen Xanthomonas oryzae pv. oryzae
Source: PLoS One. 2026 Apr 24;21(4):e0346082. doi: 10.1371/journal.pone.0346082 (PMC13108755; doi:10.1371/journal.pone.0346082)
Supplement: S1 Table — This file contains the environmental data cited in the main text. (DOCX) [file pone.0346082.s001.docx]

# S1 Table: Weather data during the growing season (July–November 2023)

| Months | Mean Temperature (°C) | Relative Humidity (%) | Total Rainfall (mm) | Climatic Condition |
| --- | --- | --- | --- | --- |
| July | 30–33 (avg. ~31) | 78–85 | 420–430 | Peak monsoon |
| August | 29–32 (avg. ~30) | 75–82 | 330–360 | Monsoon |
| September | 28–31 (avg. ~29) | 70–80 | 250–280 | Late monsoon |
| October | 26–30 (avg. ~28) | 65–75 | 120–150 | Post-monsoon |
| November | 22–27 (avg. ~25) | 55–65 | 20–40 | Dry season onset |
