# Supplementary material for: Green synthesis and characterization of zinc chitosan nanoparticles with their anti-bacterial study against rice pathogen Xanthomonas oryzae pv. oryzae
Source: PLoS One. 2026 Apr 24;21(4):e0346082. doi: 10.1371/journal.pone.0346082 (PMC13108755; doi:10.1371/journal.pone.0346082)
Supplement: Attachment — Submitted filename: Author Response letter 2026.docx [file pone.0346082.s003.docx]

**Response to Reviewers comments:**

**Title of the Manuscript:** **Green synthesis and characterization of zinc chitosan nanoparticles with their anti-bacterial study against rice pathogen *Xanthomonas oryzae* pv. *oryzae***

**Submission ID: PONE-D-25-47822**

Dear Editor,

We sincerely appreciate the opportunity to resubmit our manuscript to your esteemed journal. We are grateful for your careful review and the thoughtful editorial comments, which have significantly contributed to the improvement of our work. Your guidance has been instrumental in refining the manuscript, and we have made comprehensive revisions in response to reviewers suggestions. Below, we provide a detailed, point-by-point response regarding declaration section.

**Reviewer number 1 comments**

**Comments to the Author**

1. abstract need to be more dramatic narration.

**Response:**

We appreciate this valuable suggestion. The Abstract has been revised to be more concise and to emphasize the key objectives, novelty, and principal findings of the study, using a more engaging and less conclusion-driven narrative. This revision is intended to clearly convey the significance and relevance of the work at a glance. Changes can be seen in track change. Please see line number 86-94.

2. Fif 6 of XRD need to be separated to two figures.

**Response:** Thanks for your nice observation to make Figure 6 into two XRD figures. With due

respect, the Figure 6 cannot be separatable in the current format, as the data are derived from a single experimental dataset and are intended to be interpreted collectively for accurate comparison.

3. figure 9,10 the axis y is lesion length and its better to but the subtitles of axis x more abbreviate and explain it on the caption.

**Response:**

We agree that clarity can be enhanced. For Figures 9 and 10, the axis labels have been revised: the Y-axis label is now standardized to "Lesion Length (mm)," and the X axis subtitles (treatment groups) have been abbreviated for visual clarity. A comprehensive explanation of these abbreviations has been added to the respective figure captions. Changes can be visible in track change. Please see figure number 9 and 10 in the revised version.

**Reviewer number 2 comments**

1. All Xoo in the text should be italicized

**Response:**

Thank you for the insightful observations. All occurrences of Xoo in the text have now been italicized to maintain correct scientific formatting and consistency throughout the document. Changes can be seen in track change in the revised manuscript.

2. The Introduction section is too lengthy, authors should DELETE L135- L158 and L170 – L175

**Response:**

Thank you for the valuable suggestion. We agree that the Introduction section was overly lengthy. As recommended, we have deleted the content from L150–L 173 and 185-190 to improve clarity, focus, and overall readability of the manuscript. Changes can be visible in track change.

3. In the justification, only zinc oxide was stated to be biosynthesized, CHECK. The justification need to be rewritten to cover the scope of the study

**Response:**

Thank you for the observation. We have made the necessary corrections as per your recommendation/suggestions, and the revisions will be visible in the tracked changes. Please see line number 220-224.

4. The FTIR analysis was conducted for only ZnNPs, L261 – L266?

**Response:**

Thank you for your observation. We have corrected the term to ZncHNPs instead of ZnNPs.

5. L305 – L307, L398, Xoo was incubated for 21 days and measured at 21 days? CHECK

**Response:**

Thank you for pointing this out. Yes, the incubation period was 21days but data collected at 24 hours, 48 hours, 7 days, and 21 days intervals.

6. L395, the in vitro experiment was with the causal pathogen not the disease, CORRECT

**Response:**

We thank the reviewer for this valuable comment. We fully agree with the suggestion and have revised the manuscript accordingly to improve clarity and scientific rigor. The relevant changes have been incorporated in the revised version. Changes can be visible in track change.

7. Fig 8 legend is not in agreement with the Methodology and Results of the in vitro experiment.

**Response:**

We apologize for the inconsistency. The legend for Figure 8 has been thoroughly reviewed and revised to ensure it accurately and completely describes the methodology and results of the in vitro experiment, maintaining full agreement with the corresponding text sections. Changes can be visible in track change.

8. L321, what concentration of Xoo was used?

**Response:**

Thank you for your comment. We appreciate the clarification request regarding L321. The concentration of *Xoo (*1 × 10^8^ CFU mL^-1^ ) used in the experiment has now been clearly specified in the revised manuscript, and the correction is visible in the tracked changes. Changes can be visible in track change.

9. L322 – L323, foliar spray of what?

**Response:**

Thanks for your nice observation. We appreciate the need for clarity. We have specified at L322-L323 (and in the relevant Materials and Methods section) that the foliar spray consisted of the prepared ZnChNP working suspension at various tested concentrations and ratios, as detailed in the methodology. Changes can be visible in track change.

10. The in vivo experimental design of the treatment is not scientifically acceptable. How will the authors compare Bismerthizol (0.2%) to a lower concentration range in combination with the synthesized NPs.

**Response:**

This is a crucial point, and we have clarified the rationale in the revised manuscript. The comparison was designed to evaluate the synergistic effect of our biosynthesized ZnChNPs in a reduced-dose combination with the commercial standard (Bismerthizol). We are not comparing the two treatments head-to-head for equivalent efficacy, but rather demonstrating that a combination therapy using the commercial chemical at a lower, eco-friendlier concentration (0.15% in the combination group) alongside our NPs yields superior or comparable control to the recommended dose of Bismerthizol alone. This approach is justified by the aim to reduce chemical reliance while maintaining high efficacy. Changes can be visible in track change.

11. The coordinates of the field used should be reported

**Response:**

We thank the reviewer for this suggestion. The geographic location of the field experiment has now been specified in the revised manuscript. The field trials were conducted at the research fields of Bangladesh Rice Research Institute (BRRI), Joydebpur, Gazipur, Bangladesh (approximately 23.99° N, 90.41° E). Changes can be visible in track change.

12. L346 – L347, what was sprayed, concentration, quantity?

**Response:**

We appreciate your comments. The spray parameters like concentration, quantity was used in this stage were identical to those established in the net house experiment. Changes can be visible in track change.

13. How many replicates was used on the field, which plants were used for data acquisition, how was dispersal of treatments into non targeted plants avoided?

**Response:**

We thank the reviewer for these important questions. The field experiment was conducted using a randomized block design with three independent replicates per treatment. For each replicate, data were collected from pre-selected, uniformly grown plants located in the central area of each plot to minimize edge effects. To prevent unintended dispersal of treatments to non-target plants, buffer zones were maintained between plots, and nanoparticle applications were carried out using controlled, lowpressure spraying under calm weather conditions. These methodological details have now been clarified in the revised manuscript. Changes can be visible in track change.

14. L526, no biofilm experiment was carried out in this study. CHECK

**Response:**

We thank the reviewer for the careful observation. The reference to a biofilm experiment at L526 was an error during manuscript preparation. It has been now corrected/deleted in the revised manuscript. However, no such experiment was performed in this study. Changes can be visible in track change.

15. L569 – L585, no antibacterial mechanism study was carried out in this study, why discuss it? Hence, its advised that the authors carry out an in vitro antibacterial mechanism study

**Response:**

Thanks for your nice query. We acknowledge that a dedicated in vitro mechanistic study was not performed. We have now restricted the discussion to a plausible mechanistic explanation based on existing literature for similar Zn- and Chitosan-based NPs, focusing on factors like ROS generation and membrane disruption. Crucially, we have added a sentence in the Conclusion section to explicitly acknowledge this as a limitation of the current study and propose a dedicated mechanistic investigation as a direction for future research.

16. L600, in vitro? CHECK

**Response:**

We thank the reviewer for this valuable comment. The context at L600 has been checked and the term has been corrected in the revised manuscript. Changes can be visible in track change.

17. Fig 11 legend does not describe what is presented in the panel

**Response:**

We appreciate the reviewer’s insightful comment and agree with the concern raised. The suggested modification has been implemented, and the corresponding section has been revised to better reflect the intended meaning. The legend for Figure 11 has been entirely rewritten to accurately and comprehensively describe the data presented in the panel, ensuring it aligns with the Results section. Changes can be visible in track change.

**Reviewer number 3 comments**

1. Clarity and Structure of the Manuscript

o The Introduction should be condensed to focus more on the rationale and novelty of the work rather than general information on Xoo and BLB.

**Response:**

We thank the reviewer for this valuable comment. We fully agree with the suggestion and have revised the manuscript accordingly to improve clarity and scientific rigor. The relevant changes have been incorporated in the revised version. The Introduction has been condensed to emphasize the rationale and novelty of the study, with general background information on Xanthomonas oryzae pv. oryzae (Xoo) and bacterial leaf blight (BLB) reduced accordingly in the revised manuscript. Changes can be visible in track change.

o The Results section should integrate morphological and structural characterization results (e.g., SEM/TEM/XRD) into a cohesive discussion, avoiding repetition.

**Response:**

We appreciate the reviewer’s insightful comment and agree with the concern raised. The suggested modification has been implemented, and the corresponding section has been revised to better reflect the intended meaning. The Results section has been revised to integrate morphological and structural characterization findings (e.g., SEM, TEM, and XRD) into a single, cohesive discussion, with repetitive descriptions removed. Changes can be visible in track change.

2. Particle Size Discrepancy
o The size difference between SEM/TEM (81–300 nm) and DLS (up to 861 nm) suggests possible aggregation in aqueous suspension. This should be acknowledged and explained (e.g., hydrodynamic diameter, particle interaction, or chitosan coating effects).

**Response:**

We thank the reviewer for pointing this out. We agree with the comment. The observed discrepancy between particle sizes obtained from SEM/TEM (81–300 nm) and DLS measurements (up to 861 nm) has been acknowledged and clarified. This difference arises because DLS measures the hydrodynamic diameter of particles in aqueous suspension, which includes solvent layers and surface-bound chitosan, whereas SEM and TEM provide the physical core size under dry conditions. In addition, partial aggregation and interparticle interactions in solution can contribute to the larger apparent size detected by DLS. This explanation has been incorporated into the revised manuscript. Changes can be visible in track change.

3. Control Treatments
o Include a comparison with either chemically synthesized ZnO nanoparticles or chitosan-only nanoparticles under identical conditions. This will better demonstrate the advantage of the green-synthesized ZnChNPs.

**Response:**

We appreciate the reviewer’s suggestion to include a comparative analysis with chemically synthesized ZnO nanoparticles or chitosan-only nanoparticles under identical conditions. However, such comparative experiments were not feasible within the scope of the present study due to limitations in experimental design, time, and materials availability. Nevertheless, the advantages of the green-synthesized ZnChNPs have been discussed in the context of their eco-friendly synthesis route, structural stability, and functional surface chemistry, with comparisons supported by relevant literature.

4. Mechanistic Insight
o The manuscript should expand on the antibacterial mechanism of ZnChNPs against Xoo. For instance, reactive oxygen species (ROS) generation or Zn²⁺ ion release could be discussed with references to relevant literature.

**Response:**

We thank the reviewer for this valuable comment. We fully agree with the suggestion and have revised the manuscript accordingly to improve clarity and scientific rigor. We acknowledge that a specific in vitro mechanistic investigation was not conducted in this study. Accordingly, the discussion has been limited to plausible mechanistic interpretations supported by existing literature on comparable zinc- and chitosan-based nanoparticles, with emphasis on processes such as reactive oxygen species generation and membrane disruption. Importantly, this limitation has been explicitly stated in the Conclusion, along with the recommendation that a dedicated mechanistic study be undertaken in future work.

5. Field Experiment Data
o Provide environmental data (temperature, humidity, rainfall) during field trials to validate reproducibility. Also, clarify the frequency and timing of nanoparticle applications.

**Response:**

We acknowledge the reviewer’s suggestion for detailed environmental parameters during the field trials. The environmental data (temperature, humidity, rainfall) during field trials are given in supplementary Table 1.

**Reviewer number 4 comments**

1. The abstract is written in a conclusion-oriented style. It should be made more concise and focused on the key objectives, methods, and findings.

**Response:**

We thank the reviewer for pointing this out. We agree with the comment and have carefully revised the manuscript. The abstract has been thoroughly revised for conciseness and to focus on objectives, methods, and key findings. Changes can be visible in track change.

2. The research hypothesis and novelty are not clearly articulated. The authors should explicitly state how this study differs from existing work and what scientific gap it addresses.

**Response:**

We thank the reviewer for the comment. We have significantly revised the final paragraphs of the Introduction section to explicitly state the research hypothesis and clearly articulate the novelty of our work. The novelty lies in the eco-friendly green synthesis of the specific Zn-chitosan nanocomposite using tomato extract and its comprehensive evaluation against Xoo under controlled (in vitro and net house) and field conditions, which addresses the gap in sustainable, field-applicable control strategies for BLB. Changes can be visible in track change.

3. Minor corrections are required:
a) Add a full stop after reference [42].
b) Correct the sentence to “Synthesis was confirmed by UV–visible spectroscopy at 356 nm.”
c) Replace “X-ray spectroscopy” with “Energy Dispersive X-ray Spectroscopy (EDS).”
d) Correct the spelling of “supernatant.”

**Response:**

Thank you for pointing out these minor errors. All advised minor corrections have been implemented throughout the text of the revised manuscript. Changes can be visible in track change.

4. A brief discussion on the preparation and physicochemical properties of chitosan should be included to provide better context.

**Response:**

We appreciate the reviewer’s insightful comment and agree with the concern raised. A brief paragraph detailing the source, preparation, and key physicochemical properties of the chitosan used (e.g., molecular weight, degree of deacetylation) has been added to the Materials and Methods section to provide essential context for its role in the nanocomposite's synthesis and function. Changes can be visible in track change.

**Response:**

5. The crystalline size can be determined from the XRD data using the Scherrer equation. The authors may refer to and cite the following relevant studies:
o <https://doi.org/10.1016/j.sajce.2025.02.002>
o <https://doi.org/10.1016/j.inoche.2025.114481>

**Response:**

We thank the reviewer for this excellent suggestion. We have now applied the Scherrer equation to the main diffraction peaks of the XRD data and the calculated average crystalline size of the ZnChNPs has been included in the methodology section. The relevant text has been revised, and the suggested literature citations (or similar relevant papers) have been included in the reference list and cited appropriately. Changes can be visible in track change.

6. A comparative table contrasting the synthesized nanocomposite with previously reported materials should be added, supported by updated and relevant references, such as:
o <https://doi.org/10.1007/s12649-024-02773-0>
o <https://doi.org/10.1016/j.desal.2025.119437>

**Response:**

We appreciate the reviewer’s suggestion to include a comparative table contrasting the synthesized nanocomposite with previously reported materials. Unfortunately, we were unable to access the full texts of the specific publications cited (DOI: 10.1007/s12649- 024-02773-0 and DOI: 10.1016/j.desal.2025.119437) due to subscription and availability limitations.

7. The manuscript should also include a plausible mechanistic explanation of how the synthesized nanocomposite disrupts bacterial cells.

**Response:**

We thank the reviewer for this valuable comment. We fully agree with the suggestion and have revised the manuscript accordingly to improve clarity and scientific rigor. The relevant changes have been incorporated in the revised version. The Discussion section has been expanded to include a plausible, literature-grounded mechanistic explanation for the antibacterial action of the ZnChNPs. Changes can be visible in track change.

We look forward to hearing from you in due time regarding our submission and to respond to any further questions and comments you may have.

Thank you once again for your time and consideration of our manuscript.

**Dr. Mohammad Abdul Latif** (Corresponding Author)

Director (Admin and common service),

Former Head and Chief Scientific Officer,

Plant Pathology Division,

Bangladesh Rice Research Institute (BRRI)

Phone: +8801715034094

E-mail: [latifbrri@gmail.com](mailto:latifbrri@gmail.com), [alatif1965@yahoo.com](mailto:alatif1965@yahoo.com)

ORCID id: <https://orcid.org/0000-0002-4289-575X>
