# Supplementary material for: Green synthesis and characterization of zinc chitosan nanoparticles with their anti-bacterial study against rice pathogen Xanthomonas oryzae pv. oryzae
Source: PLoS One. 2026 Apr 24;21(4):e0346082. doi: 10.1371/journal.pone.0346082 (PMC13108755; doi:10.1371/journal.pone.0346082)
Supplement: Attachment — Submitted filename: Author Response letter R4.docx [file pone.0346082.s004.docx]

**Response to Reviewers**

**Title:** Green synthesis and characterization of zinc chitosan nanoparticles with their anti-bacterial study against rice pathogen *Xanthomonas oryzae* pv. *oryzae*

**Manuscript ID:** PONE-D-25-47822R1

Dear Editor,

We sincerely thank you and the reviewers for your careful evaluation of our manuscript and for the constructive feedback provided. We are pleased that the reviewers found the revised manuscript significantly improved and technically sound. We have carefully addressed all remaining comments and made the requested revisions accordingly.

All changes in the manuscript are highlighted in the “Revised Manuscript with Track Changes” file. Below, we provide a detailed point-by-point response.

**Responses to Reviewer #1**

**Comment:**
The manuscript is significantly improved, the authors addressed all the comments needed, and the manuscript in its current form is suitable for publication.

**Response:**
We sincerely thank the reviewer for the positive evaluation and for acknowledging the improvements made in the revised manuscript. We appreciate the reviewer’s time and valuable feedback, which greatly enhanced the quality and clarity of our work.

**Responses to Reviewer #2**

**Comment 1:**

All acronyms in the abstract should be defined at first mention e.g. FESEM, SEM, TEM, EDS, FTIR, XRD, BLB, etc.

**Response:**

Thank you for this important suggestion. All acronyms in the abstract have now been defined at their first mention. Specifically:

- FESEM – Field Emission Scanning Electron Microscopy
- SEM – Scanning Electron Microscopy
- TEM – Transmission Electron Microscopy
- EDS – Energy Dispersive Spectroscopy
- FTIR – Fourier Transform Infrared Spectroscopy
- XRD – X-ray Diffraction
- BLB – Bacterial Leaf Blight

These corrections have been implemented in the Abstract section (See Line number 81-87) to improve clarity and readability. Changes can be seen in track change

**Comment 2:**

For the in vitro antibacterial experiment, the Xoo growth is already static at 7 and 21 days and effect of NPs are evident within 24 to 72 hours. Hence, the graphical report of Xoo at 7 and 21 days were not significant. It is advised that the authors delete the measurement at 7 and 21 days and focus on 24 and 48 hours readings which is scientifically acceptable. This correction should be effected in the Methodology, Results, and Discussion.

**Response:**
We appreciate this scientifically valuable suggestion.

Following the reviewer’s recommendation:

1. The antibacterial growth measurements at 7 and 21 days have been removed from:
   - Methodology section (See Line number 280-286)
   - Results section (See Line number 372-387)
   - Discussion section (See Line number 507-510)
   - Corresponding figures and figure legends (See Fig8)
2. The study now focuses exclusively on the 24- and 48-hour time points, where nanoparticle effects were clearly evident and biologically meaningful.
3. The statistical analysis has been revised accordingly to reflect only the 24- and 48-hour data.
4. Figures have been updated to present only scientifically relevant time points, improving clarity and data interpretation.

These revisions enhance the scientific rigor and focus of the antibacterial evaluation.

We believe that the manuscript has now fully addressed all reviewer and editorial concerns. We are grateful for the constructive feedback that helped improve the scientific quality, clarity, and presentation of our work.

We sincerely thank the Academic Editor and reviewers for their time and consideration. If need any further information or improvement, please let us know. We look forward to your favorable decision.

Kind regards,

**Dr. Md. Abdul Latif**
(On behalf of all authors)
Bangladesh Rice Research Institute (BRRI)
Gazipur, Bangladesh
